# Supplementary material for: In silico genomic surveillance by CoVerage predicts and characterizes SARS-CoV-2 variants of interest
Source: Nat Commun. 2025 Jul 8;16:6281. doi: 10.1038/s41467-025-60231-4 (PMC12238648; doi:10.1038/s41467-025-60231-4)
Supplement: Supplementary file 1 — Supplementary Information [file 41467_2025_60231_MOESM1_ESM.pdf]

In silico genomic surveillance by CoVerage predicts and characterizes SARS-CoV-2 Variants of Interest

Supplementary Information

Provided in this supplementary material is an example of the CoVerage homepage (**Supplementary Fig. 1**), comparison of antigenic weights derived from influenza A/H3N2 antigenic and genetic data and the corresponding changes in amino acid properties (**Supplementary Fig. 2**), the results of a comparison between predicted antigenic alteration scores and averaged EVEscape scores for VOCs circulating between January 2020 to December 2023 (**Supplementary Fig. 3**), an allele dynamics plot for the USA (**Supplementary Figure 4**), and the averaged standardized antigenic alteration scores of WHO designated VOC lineages and non-designated lineages (**Supplementary Figure 5**). Additionally, the reference material used in the variant antigenic alteration scoring analysis (**Supplementary Table 1**) and the statistical results for the Spearman's Correlation and one-sided Wilcoxon sign-rank test comparing correlation of different scoring methods to ground truth such as antigenic distances and averaged mFRN values (**Supplementary Table 2-7**) are provided. The circulating variants for the month of March 2023 are given with their associated antigenic alteration scores and ranked from highest to lowest for that month. Also included are the results for the lineage dynamics analysis for the months of January 2023 through March 2023 (**Supplementary Tables 8-10**). These tables list the selected pVOIs and their median p-value along with their WHO designation as either a variant under monitoring (VUM) or a variant of concern (VOC).

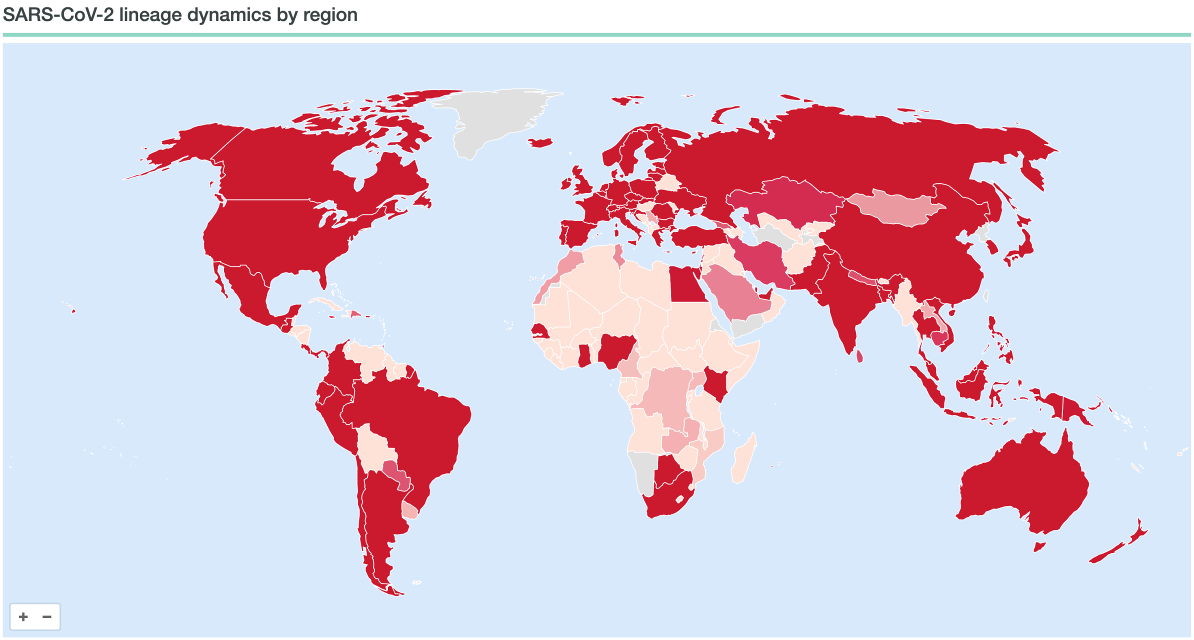

Supplementary Fig. 1: Global map representing the SARS-CoV-2 lineage dynamics by region on the CoVerage homepage. Countries shown in red have more than 2000 sequences and have the lineage dynamics analysis results available. Case numbers per country are obtained from the WHO Coronavirus (COVID-19) data repository[19].

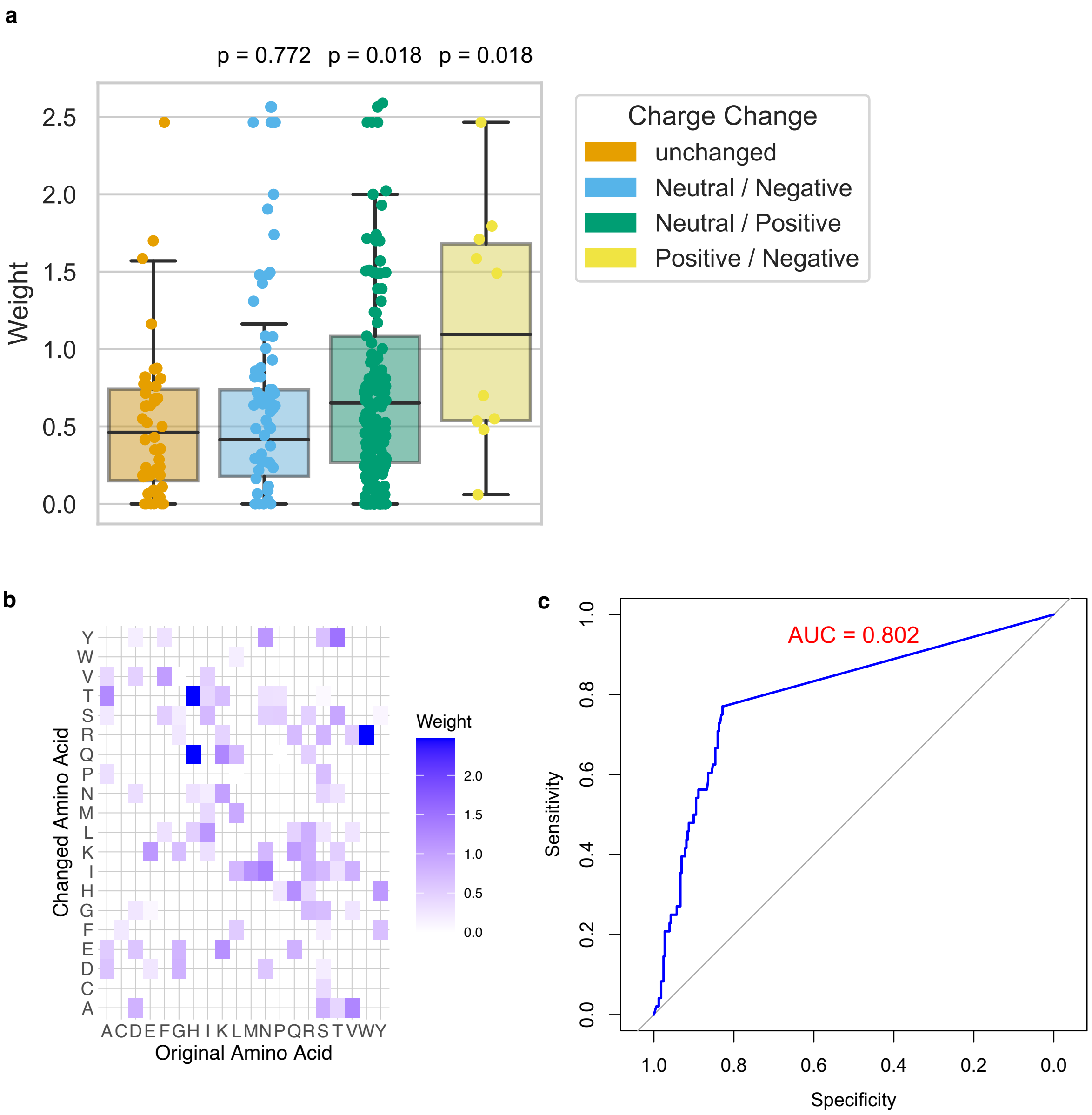

Supplementary Figure 2: a Boxplot of the antigenic alteration weights (with arbitrary units) for amino acid changes in antigenic drift of human influenza A/H3N2 viruses[64] grouped into different charge categories: neutral / negative (n = 47) representing changes from a neutral to a negative amino acid or vice versa, neutral / positive (n = 60) representing changes from a neutral to a positive amino acid and vice versa, positive / negative (n = 10) representing a change from a positive to a negative amino acid and vice versa, and the unchanged category (n = 190), where the amino acid charge stays the same. The FDR corrected p-values (with Benjamini Hochberg procedure) from the Wilcoxon rank sum test comparing the antigenic weights of the amino acid change property groups to the antigenic weights of the unchanged group are shown above the corresponding box and whisker plot. The horizontal bar in each box represents the median value of the group, the top of the box represents the 25th or upper quartile (Q1), the bottom of the box the lower quartile 75th (Q3) and the whiskers extend to the smallest and largest values within 1.5 times the interquartile range (Q1 - Q3) with the points beyond the whiskers representing outliers. **b** Heatmap of amino acid changes and their antigenic alteration weights used for scoring antigenic alterations of SARS-Cov-2 lineages derived from the influenza A/H3N2 antigenic and genetic data[64]. Dark purple represents a higher antigenic alteration weight and a light purple represents a lower antigenic alteration weight, with arbitrary units. Blank spaces in the heatmap represent amino acid changes that do not have a weight. **c** ROC curve assessing the predictive power of antigenic

alteration weights for specific amino acid alterations for changes altering the antigenicity for SARS-CoV-2. The AUC is given in red (AUC of 0.802).

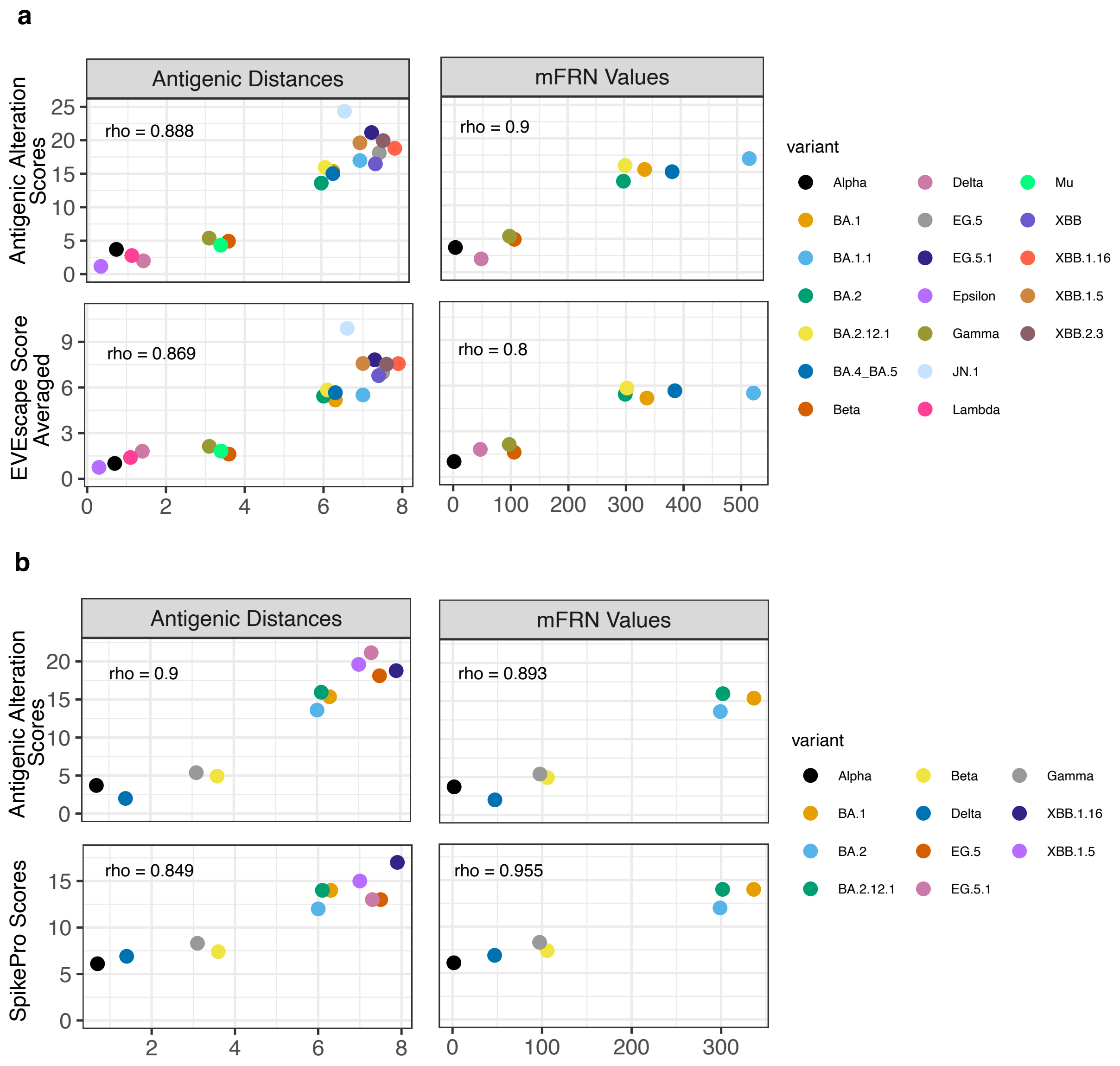

Supplementary Fig. 3: Comparison of predicted antigenic alteration scores and averaged EVEscape scores for VOCs circulating between January 2020 to December 2023 to a antigenic distances from both human and hamster sera (n=19) and the averaged mFRN values (n=9) (both with arbitrary units). **b** Comparison of predicted antigenic alteration scores and SpikePro scores for VOCs circulating between January 2020 and December 2023 against the antigenic distances (n=11) and mFRN values (n=7) (both with arbitrary units).

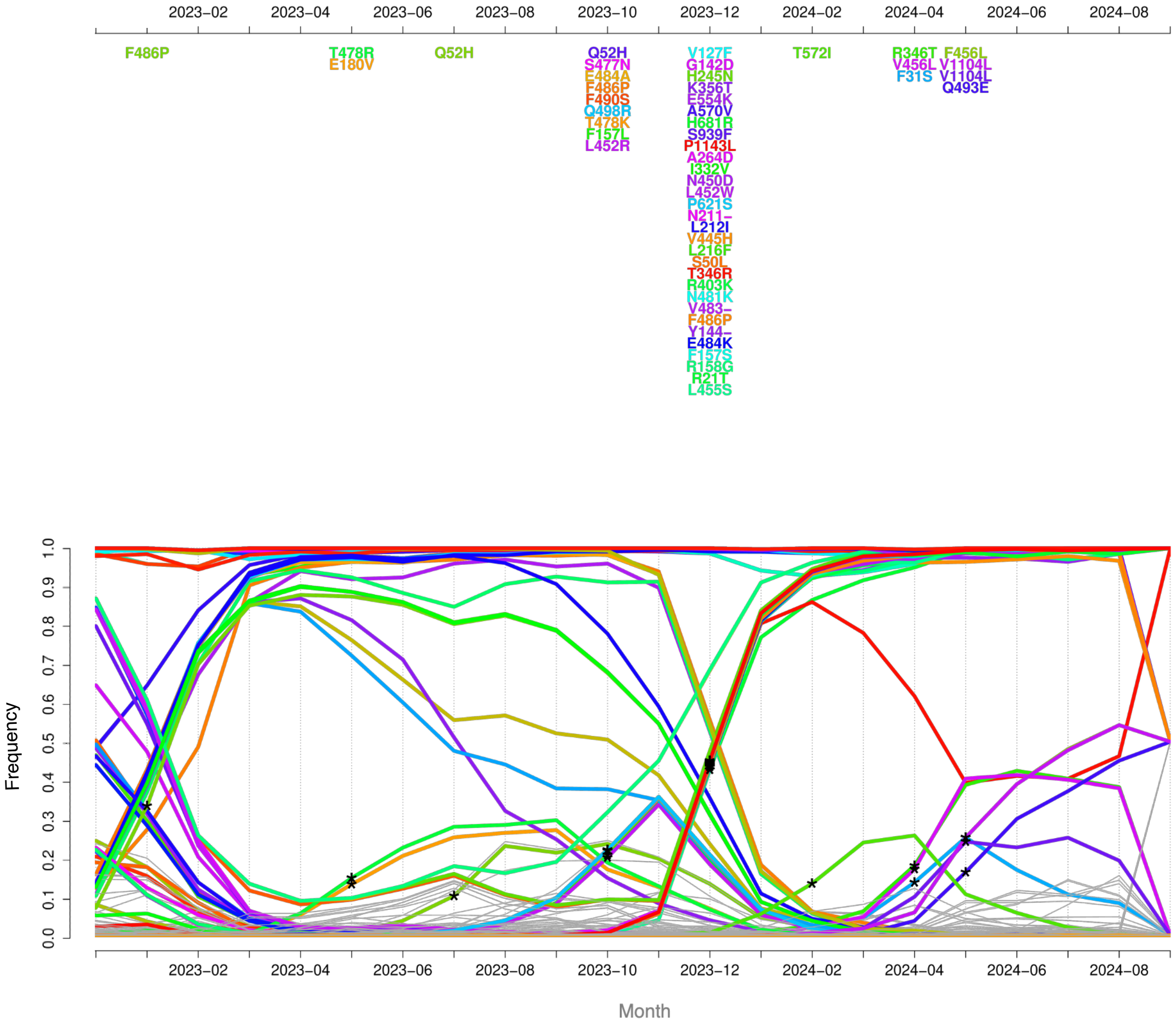

Supplementary Fig. 4: SD plot for United States of America from December 2022 to July 2024. The asterisks represent when a spike protein allele with certain amino acid changes significantly rises in frequency and the color of the curve corresponds to the associated amino acid change. Amino acid changes are specified at the top of the plot, based on the time point when they were identified as significant.

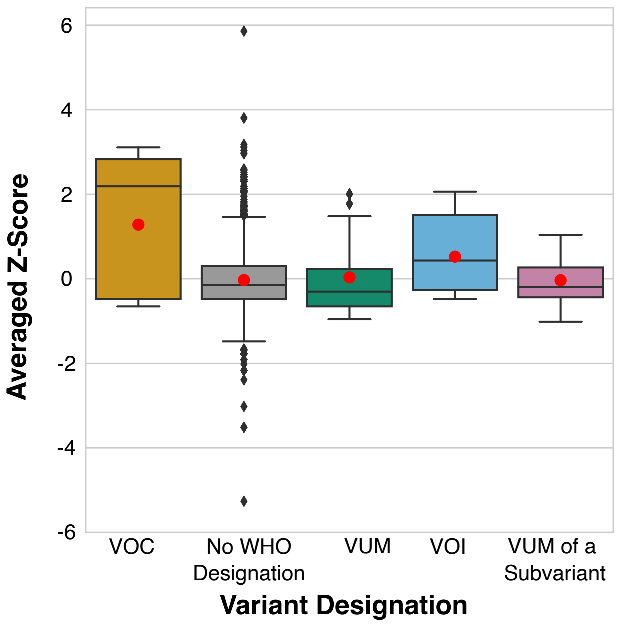

Supplementary Fig. 5: Averaged standardized antigenic alteration scores (z-scores) of WHO designated Variant of Concern (VOC), Variant of Interest (VOI) and Variant Under Monitoring VUM of a subvariant, and non-designated lineages from January 2020 to December 2023 (calculated from a total of 16,204,027 sequences). The upper quartile is given as the top of the box, the median as the middle line, and the lower quartile as the bottom of the box. The whiskers extend to the smallest and largest values within 1.5 times the interquartile range with the points beyond the whiskers representing the outliers of the group. The red dot represents the mean of the respective designation.

Supplementary Table 1: Selected amino acid sites in the SARS-CoV-2 spike protein that have been shown to alter SARS-CoV-2 antigenicity using literature available up to June 30, 2021.

antigenic_sites	information	Reference
18	escape from NTD-binding mAbs, reduces antibody neutralization	Harvey et al., 2021 (https://doi.org/10.1038/s41579-021-00573-0); McCallum et al., 2021(10.1016/j.cell.2021.03.028)
157	reduces neutralization by mAb (2489)	Harvey et al., 2021 (https://doi.org/10.1038/s41579-021-00573-0); Suryadevara et al., 2021 (10.1016/j.cell.2021.03.029)
234	resistant to neutralizing antibodies	Li et al., 2020 (https://doi.org/10.1016/j.cell.2020.07.012)
417	escape neutralization by mAbs, enhanced binding with ACE2	Starr et al., 2021 (DOI: 10.1126/science.abf9302)
439	increased binding affinity for ACE2 through formation of new salt bridge, resistant to some neutralizing antibodies	Harvey et al., 2021 (https://doi.org/10.1038/s41579-021-00573-0); Li et al., 2021 (https://doi.org/10.1038/s41392-021-00592-6)
440	immune escape; reduced the neutralization by mAbs	Starr et al., 2021 (DOI: 10.1126/science.abf9302)
444	immune escape the neutralization by mAbs and human convalescent sera	Harvey et al., 2021 (https://doi.org/10.1038/s41579-021-00573-0); Liu et al., 2021 (10.1016/j.chom.2021.01.014); Starr et al., 2021 (DOI: 10.1126/science.abf9302)
446	immune escape the neutralization by mAbs and human convalescent sera	Harvey et al., 2021 (https://doi.org/10.1038/s41579-021-00573-0); Liu et al., 2021 (10.1016/j.chom.2021.01.014)
450	immune escape the neutralization by mAbs and human convalescent sera	Liu et al., 2021 (10.1016/j.chom.2021.01.014)
452	reduces neutralization by mAbs, enhances infectivity	Harvey et al., 2021 (https://doi.org/10.1038/s41579-021-00573-0); Li et al., 2020 (https://doi.org/10.1016/j.cell.2020.07.012); Liu et al., 2021 (10.1016/j.chom.2021.01.014)
453	reduces neutralization by mAbs, increase ACE2 affinity	Harvey et al., 2021 (https://doi.org/10.1038/s41579-021-00573-0); Starr et al., 2021 (DOI: 10.1126/science.abf9302)
475	immune escape the neutralization by mAbs and human convalescent sera	Li et al., 2020 (https://doi.org/10.1016/j.cell.2020.07.012)
476	immune escape the neutralization by mAbs	Tortorici et al., 2020 (https://doi.org/10.1126/science.abe3354); Rogers et al., 2020 (https://doi.org/10.1126/science.abc7520)
477	immune escape, resistant to neutralization, enhanced binding with ACE2	Harvey et al., 2021 (https://doi.org/10.1038/s41579-021-00573-0); Liu et al., 2021 (https://doi.org/10.1101/2020.11.06.37

		2037); Liu et al., 2021 (10.1016/j.chom.2021.01.014)
478	immune escape the neutralization by mAbs and human convalescent sera	Liu et al., 2021 (10.1016/j.chom.2021.01.014)
479	immune escape the neutralization by mAbs	Liu et al., 2021 (10.1016/j.chom.2021.01.014)
483	resistant to some neutralizing antibodies and to human convalescent sera	Li et al., 2020 (https://doi.org/10.1016/j.cell.2020.07.012)
484	reduces neutralization of antibodies, immune escape	Harvey et al., 2021 (https://doi.org/10.1038/s41579-021-00573-0); Liu et al., 2021 (10.1016/j.chom.2021.01.014)
486	immune escape the neutralization by mAbs	Liu et al., 2021 (10.1016/j.chom.2021.01.014)
489	immune escape the neutralization by mAbs	Starr et al., 2021 (DOI: 10.1126/science.abf9302)
490	resistant to some neutralizing antibodies	Liu et al., 2021 (10.1016/j.chom.2021.01.014)
493	immune escape the neutralization by mAbs	Liu et al., 2021 (10.1016/j.chom.2021.01.014); Starr et al., 2021 (DOI: 10.1126/science.abf9302)
499	immune escape the neutralization by mAbs and human convalescent sera	Liu et al., 2021 (10.1016/j.chom.2021.01.014)
501	reduces neutralization of RBD antibodies, increases ACE2 binding affinity, increases transmissibility	Harvey et al., 2021 (https://doi.org/10.1038/s41579-021-00573-0); Starr et al., 2021 (DOI: 10.1126/science.abf9302)
681	immune escape the neutralization by mAbs and human convalescent sera	Saito et al., 2021 (https://doi.org/10.1101/2021.06.17.448820)
769	decreased susceptibility to neutralizing antibodies	Harvey et al., 2021 (https://doi.org/10.1038/s41579-021-00573-0)
796	reduction in susceptibility to non-RBD specific antibodies, but decreases infectivity	Kemp et al., 2021 (https://doi.org/10.1038/s41586-021-03291-y)

Supplementary Table 2: Results from the two-sided Spearman's correlation comparing the various antigenic scoring methods' predicted scores of known VOCs to antigenic cartography distances and mFRN values. The adjusted p-value is given after Benjamini-Hochberg FDR correction (alpha = 0.05)[22]. In comparing the antigenic scores to the antigenic distances there were a total of 19 samples, and for mFRN values a total of 9 samples.

**Spearman's Correlation Comparing Antigenic Scores to Antigenic Cartography Distances**				
**Antigenic Scoring Method**	**rho correlation value**	**Test Statistic (S)**	**p-value**	**Adjusted p-value**
Without weights at antigenic sites	0.833553	189.75	0.000009304033	0.00001395605
Weights at all sites	0.888499	127.11	0.0000003755082	0.000001278612
Weights at antigenic sites	0.6874454	356.31	0.001144530	0.001144530
Counting all amino acid changes	0.8481127	173.15	0.000004502551	0.000009005102
Weights without directionality at all sites	0.8867431	129.11	0.0000004262041	0.000001278612
Higher threshold weights at all sites	0.791923	237.21	0.00005333581	0.00006400297
**Spearman's Correlation Comparing Antigenic Scores to Average mFRN Values**				
**Antigenic Scoring Method**	**rho correlation value**	**Test Statistic (S)**	**p-value**	**Adjusted p-value**
Without weights at antigenic sites	0.8166667	22	0.0072247852	0.007224785
Weights at all sites	0.9	12	0.0009430623	0.001414593
Weights at antigenic sites	0.85	18	0.0037047773	0.004445733
Counting all amino acid changes	0.9	12	0.0009430623	0.001414593
Weights without directionality at all sites	0.9166667	10	0.0005066191	0.001414593
Higher threshold weights at all sites	0.9	12	0.0009430623	0.001414593

Supplementary Table 3: Results from the one-sided Wilcoxon signed-rank test comparing the deviations of normalized methods (method 2 where antigenic weights were applied at all amino acid changes on the spike protein and the baseline method which counted amino acid changes on the spike protein) from the normalized ground truth antigenic cartography distances (n=19). Utilizing the normalized deviations allows for the comparison of how closely the tested method correlates to the ground truth antigenic distances in comparison to the baseline method. The results of each method were normalized using min-max normalization and then compared to the normalized ground truth values, ie. the antigenic distances using the Wilcoxon signed-rank test (R wilcox.test; alternative = "less", exact = FALSE, correct = TRUE, paired = TRUE).

VOC	Scaled Antigenic Cartography Distances	Scaled Antigenic Scores (Weights at all sites)	Deviation	Scaled Antigenic Scores (Counting all amino acid changes)	Deviation
Alpha	0.05263158	0.10962451	0.05699294	0.11249767	0.05986609
Beta	0.43421053	0.16227881	0.27193172	0.11007636	0.32413416
Gamma	0.36842105	0.18213207	0.18628899	0.14099460	0.22742645
Epsilon	0.00000000	0.00000000	0.00000000	0.00000000	0.00000000
Delta	0.14473684	0.03582218	0.10891466	0.08809834	0.05663850
Lambda	0.10526316	0.06991800	0.03534516	0.18662693	0.08136377
Mu	0.40789474	0.13638325	0.27151148	0.10001863	0.30787611
BA.1	0.78947368	0.61286146	0.17661223	0.55280313	0.23667056
BA.1.1	0.88157895	0.68277946	0.19879949	0.58986776	0.29171119
BA.2	0.75000000	0.53690117	0.21309883	0.45203949	0.29796051
BA.2.12.1	0.76315789	0.63832542	0.12483247	0.52598249	0.23717540
BA.4_BA.5	0.78947368	0.59818731	0.19128637	0.52710002	0.26237367
XBB	0.93421053	0.66076823	0.27344229	0.52635500	0.40785553
XBB.1.16	1.00000000	0.76089771	0.23910229	0.69007264	0.30992736
XBB.1.5	0.88157895	0.79628830	0.08529064	0.67982865	0.20175030
XBB.2.3	0.96052632	0.81096245	0.14956386	0.67926988	0.28125643
EG.5	0.94736842	0.73198101	0.21538741	0.63978394	0.30758448
EG.5.1	0.92105263	0.86275356	0.05829907	0.71130564	0.20974699
JN.1	0.82894737	1.00000000	0.17105263	1.00000000	0.17105263
**Wilcoxon Signed-Rank Test:** V test statistic = 6, p-value = 0.0004604047					

Supplementary Table 4: Results from the two-sided Spearman's correlation comparing the antigenic scoring method's predicted scores of known VOCs and the EVEscape predicted fitness scores to antigenic cartography distances (n=19) and mFRN values (n=9).

Spearman's Correlation Comparing Antigenic Scores to Antigenic Cartography Distances		
**Antigenic Scoring Method**	**rho correlation value**	**p-value**
Antigenic Scoring Method	0.888499	0.0000003755
EVEscape Fitness Scores	0.8691838	0.000001364
**Spearman's Correlation Comparing Antigenic Scores to Average mFRN values**		
**Antigenic Scoring Method**	**rho correlation value**	**p-value**
Antigenic Scoring Method	0.9	0.0009431
EVEscape Fitness Scores	0.8	0.009628

Supplementary Table 5: Results of the one-sided Wilcoxon signed-rank test comparing the deviations of normalized antigenic scores of the selected method (where antigenic weights were applied at all amino acid changes on the spike protein) and the normalized EVEscape scores from the normalized ground truth antigenic cartography distances (n=19). Utilizing the normalized deviations allows for the comparison of how closely the tested method correlates to the ground truth antigenic distances in comparison to the baseline method. The results of the selected method and the EVEscape scores, averaged across January 2020 to December 2023, were normalized using min-max normalization and then compared to the normalized ground truth values, ie. the antigenic distances using the Wilcoxon signed-rank test (R wilcox.test; alternative = "less", exact = FALSE, correct = TRUE, paired = TRUE).

VOC	Scaled Antigenic Cartography Distances	Scaled Antigenic Scores (Weights at all sites)	Deviation	Scaled EVEscape Scores	Deviation
Alpha	0.05263158	0.10962451	0.05699294	0.02844639	0.02418519
BA.1	0.78947368	0.61286146	0.17661223	0.48468271	0.30479097
BA.1.1	0.88157895	0.68277946	0.19879949	0.52078775	0.36079120
BA.2	0.75000000	0.53690117	0.21309883	0.51203501	0.23796499
BA.2.12.1	0.76315789	0.63832542	0.12483247	0.55689278	0.20626512
BA.4_BA.5	0.78947368	0.59818731	0.19128637	0.53719912	0.25227456
Beta	0.43421053	0.16227881	0.27193172	0.09518600	0.33902453
Delta	0.14473684	0.03582218	0.10891466	0.11597374	0.02876310
EG.5	0.94736842	0.73198101	0.21538741	0.68161926	0.26574917
EG.5.1	0.92105263	0.86275356	0.05829907	0.77352298	0.14752966
Epsilon	0 00000000	0.00000000	0.00000000	0.00000000	0.00000000
Gamma	0.36842105	0.18213207	0.18628899	0.15098468	0.21743637
JN.1	0.82894737	1.00000000	0.17105263	1.00000000	0.17105263
Lambda	0.10526316	0.06991800	0.03534516	0.07111597	0.03414718
Mu	0.40789474	0.13638325	0.27151148	0.11706783	0.29082690
XBB	0.93421053	0.66076823	0.27344229	0.65973742	0.27447311
XBB.1.16	1.00000000	0.76089771	0.23910229	0.74617068	0.25382932
XBB.1.5	0.88157895	0.79628830	0.08529064	0.74726477	0.13431418
XBB.2.3	0 96052632	0.81096245	0.14956386	0.74179431	0.21873201
**Wilcoxon Signed-Rank Test:** V test statistic = 22, p-value = 0.00529					

Supplementary Table 6: Results from the two-sided Spearman's correlation comparing the antigenic scoring method's predicted scores of known VOCs and the SpikePro predicted fitness scores to antigenic cartography distances (n=11) and mFRN values (n=7).

Spearman's Correlation Comparing Antigenic Scores to Antigenic Cartography Distances		
**Antigenic Scoring Method**	**rho correlation value**	**p-value**
Antigenic Scoring Method	0.9	0.00016
SpikePro Fitness Scores	0.8493239	0.000938

Supplementary Table 7: Results of the one-sided Wilcoxon signed-rank test comparing the deviations of normalized antigenic scores of the selected method (where antigenic weights were applied at all amino acid changes on the spike protein) and the normalized SpikePro scores from the normalized ground truth antigenic cartography distances (n=11). Utilizing the normalized deviations allows for the comparison of how closely the tested method correlates to the ground truth antigenic distances in comparison to the baseline method. The results of the selected method and the SpikePro scores, averaged across January 2020 to December 2023, were normalized using min-max normalization and then compared to the normalized ground truth values, ie. the antigenic distances using the Wilcoxon signed-rank test (R wilcox.test; alternative = "less", exact = FALSE, correct = TRUE, paired = TRUE).

VOC	Scaled Antigenic Cartography Distances	Scaled Antigenic Scores (Weights at all sites)	Deviation	Scaled SpikePro Scores	Deviation
Alpha	0.00000000	0.08924843	0.08924843	0.0000000	0.00000000
BA.1	0.77777778	0.69780793	0.07996984	0.7247706	0.05300714
BA.2	0.73611111	0.60594990	0.13016122	0.5412844	0.19482671
BA.2.12.1	0.75000000	0.72860125	0.02139875	0.7247706	0.02522936
Beta	0.40277778	0.15292276	0.24985502	0.1192661	0.28351172
Delta	0.09722222	0.00000000	0.09722222	0.0733945	0.02382773
EG.5	0.94444444	0.84185804	0.10258641	0.6330275	0.31141692
EG.5.1	0.91666667	1.00000000	0.08333333	0.6330275	0.28363914
Gamma	0.33333333	0.17693111	0.15640223	0.2018349	0.13149847
XBB.1.16	1.00000000	0.87682672	0.12317328	1.0000000	0.00000000
XBB.1.5	0.87500000	0.91962422	0.04462422	0.8165138	0.05848624
**Wilcoxon Signed-Rank Test:** V test statistic = 31, p-value = 0.447					

Supplementary Table 8: Selected pVOIs listed by their Pango lineage for January 2023 and their median p-values as per the lineage dynamics analysis. Listed p-values result from the one-sided Fisher's exact test to determine lineages significantly on the rise in frequency. P-values were corrected using the Benjamini–Hochberg false discovery rate. Also shown is the date and designation of lineages as a VOI or Variant Under Monitoring (VUM).

Pango lineage	Median p-value	Identified as a VOC / VOI / VUM	Notes (dates given as DD-MM-YYYY)	References
BA.1.1	9.60E-63			
BA.2	4.94E-55		de-escalated VOC as per the ECDC	https://www.ecdc.europa.eu/en/covid-19/variants-concern
BA.2.10.1	0.005404522			
BA.2.3	9.27E-26			
BA.2.3.20	6.39E-67			
BA.4.6	9.11E-11		enhanced neutralization resistance respective of parental BA.4/5 subvariant	Qu. P et al., 2023
BA.5	2.21E-28		de-escalated VOC as per the ECDC, broadly resistant to most nAbs	Cao et al., 2022; https://www.ecdc.europa.eu/en/covid-19/variants-concern
BA.5.1	7.16E-63			
BA.5.11	7.41E-05			
BA.5.2	6.44E-15			
BA.5.2.1	3.25E-10			
BA.5.2.6	1.48E-09			
BE.1.1	3.08E-66			
BE.9	1.60E-65			
BF.5	7.59E-280			
BF.7	2.41E-12		enhanced neutralization resistance respective of parental BA.4/5 subvariant	Qu. P et al., 2023
BF.7.14	6.84E-31			
BN.1.2	1.97E-15			
BN.1.3	2.69E-16			
BQ.1	5.46E-14	VOI (20-10-2022)	VOI as per ECDC (20-10-2022); enhanced neutralization resistance respective of parental BA.5 subvariant	Qu. P et al., 2023; https://www.ecdc.europa.eu/en/publications-data/spread-sars-cov-2-omicron-variant-sub-lineage-bq1-eueea
BQ.1.1	3.25E-25		resistant to all clinical mAbs / enhanced neutralization resistance respective of parental BA.5 subvariant	Arora P., et al. 2022; Qu P., et al., 2023
BQ.1.1.1	3.06E-08			
BQ.1.1.10	0.003182437			
BQ.1.1.20	6.32E-82			
BQ.1.1.22	3.55E-06			
BQ.1.1.38	0.000723485			
BQ.1.1.4	0.031009802			
BQ.1.10	1.80E-05			
BR.2.1	6.67E-159			

CH.1.1	0.026651959	VUM (08-02-2023)	WHO variant under monitoring since (08-02-2023)	https://www.who.int/activities/tracking-SARS-CoV-2-variants
CH.1.1.1	0.000159952			
CH.1.1.7	7.98E-18			
CK.1	1.18E-05			
CL.1	5.48E-95			
CM.12	2.29E-07			
DY.2	5.38E-08			
DY.4	5.12E-15			
Unassigned	0.029477699			
XBB	1.42E-58	VUM (12-10-2022)	XBB and its sublineages have been identified as variants under monitoring as per the WHO (12-10-2022)	https://www.who.int/activities/tracking-SARS-CoV-2-variants
XBB.1	1.60E-05			
XBB.1.15	0.002277383			
XBB.1.5	4.52E-22	VOI (11-01-2023)	WHO current variant of interest (as of 11-01-2023)	https://www.who.int/activities/tracking-SARS-CoV-2-variants
XBB.1.5.12	2.84E-05			
XBB.1.9.1	3.02E-05	VUM (30-03-2023)	WHO variant under monitoring since (30-03-2023)	https://www.who.int/activities/tracking-SARS-CoV-2-variants
XBB.1.9.2	2.18E-07	VUM (26-04-2023	WHO variant under monitoring since (26-04-2023)	https://www.who.int/activities/tracking-SARS-CoV-2-variants
XBB.2	8.09E-58			
XBB.2.6	1.24E-115			
XBF	2.68E-08		increased antibody neutralization	Ackerman A., et al. 2023

Supplementary Table 9: Selected pVOIs listed by their Pango lineage for February 2023 and their median p-values as per the lineage dynamics analysis. Listed p-values are the result of the one-sided Fisher's exact test to determine lineages significantly on the rise in frequency. P-values were corrected using the Benjamini–Hochberg false discovery rate. Also shown is the date and designation of lineages as a VOI or Variant Under Monitoring (VUM).

Pango lineage	Median p-value	Identified as a VOC / VOI / VUM	Notes (dates given as DD-MM-YYYY)	References
BA.2	4.94E-55		de-escalated VOC as per the ECDC	https://www.ecdc.europa.eu/en/covid-19/variants-concern
BA.2.10.1	0.005404522			
BA.2.75	1.78E-200	VUM (06-07-2022)	WHO variant under monitoring since (06-07-2022), VOI as per the ECDC	https://www.who.int/activities/tracking-SARS-CoV-2-variants; https://www.ecdc.europa.eu/en/covid-19/variants-concern
BA.4.1.9	0.012054399			
BA.5.2	6.44E-15			
BA.5.2.1	3.25E-10			
BA.5.2.6	1.48E-09			
BE.9	1.60E-65			
BF.7	2.41E-12		enhanced neutralization resistance respective of parental BA.4/5 subvariant	Qu. P et al., 2023
BF.7.14	6.84E-31			
BN.1.2	1.97E-15			
BN.1.3	2.69E-16			
BQ.1	5.46E-14	VOI (20-10-2022)	VOI as per ECDC (20-10-2022); enhanced neutralization resistance respective of parental BA.5 subvariant	Qu. P et al., 2023; https://www.ecdc.europa.eu/en/publications-data/spread-sars-cov-2-omicron-variant-sub-lineage-bq1-eueea
BQ.1.1	3.25E-25		resistant to all clinical mAbs / enhanced neutralization resistance respective of parental BA.5 subvariant	Arora P., et al. 2022; Qu P., et al., 2023
BQ.1.1.1	3.06E-08			
BQ.1.1.10	0.003182437			
BQ.1.1.4	0.031009802			
BQ.1.10	1.80E-05			
BR.2.1	6.67E-159			
CH.1.1	0.026651959	VUM (08-02-2023)	WHO variant under monitoring since (08-02-2023)	https://www.who.int/activities/tracking-SARS-CoV-2-variants
CH.1.1.1	0.000159952			
CH.1.1.7	7.98E-18			
CK.1	1.18E-05			
CL.1	5.48E-95			
CM.12	2.29E-07			
DY.2	5.38E-08			

DY.4	5.12E-15			
EG.1	2.85E-08			
FL.2	1.08E-22			
FR.1	0.020160177			
Unassigned	0.029477699			
XBB	1.42E-58	VUM (12-10-2022)	XBB and its sublineages have been identified as variants under monitoring as per the WHO (12-10-2022)	https://www.who.int/activities/tracking-SARS-CoV-2-variants
XBB.1	1.60E-05			
XBB.1.15	0.002277383	VOI (11-01-2023)	WHO current variant of interest (as of 11-01-2023)	https://www.who.int/activities/tracking-SARS-CoV-2-variants
XBB.1.16	5.97E-10	VOI (17-04-2023)	WHO current variant of interest (as of 17-04-2023)	https://www.who.int/activities/tracking-SARS-CoV-2-variants
XBB.1.16.1	0.006501881			
XBB.1.18.1	2.35E-22			
XBB.1.5	4.52E-22			
XBB.1.5.12	2.84E-05			
XBB.1.5.25	3.96E-41			
XBB.1.5.33	5.67E-05			
XBB.1.9.1	3.02E-05	VUM (30-03-2023)	WHO variant under monitoring since (30-03-2023)	https://www.who.int/activities/tracking-SARS-CoV-2-variants
XBB.1.9.2	2.18E-07	VUM (26-04-2023)	WHO variant under monitoring since (26-04-2023)	https://www.who.int/activities/tracking-SARS-CoV-2-variants
XBB.2	8.09E-58			
XBB.2.6	1.24E-115			
XBF	2.68E-08		increased antibody neutralization	Ackerman A., et al. 2023

Supplementary Table 10: Selected pVOIs listed by their Pango lineage for March 2023 and their median p-values as per the lineage dynamics analysis. Listed p-values are the result of the one-sided Fisher's exact test to determine lineages significantly on the rise in frequency. P-values were corrected using the Benjamini–Hochberg false discovery rate. Also shown is the date and designation of lineages as a VOI or Variant Under Monitoring (VUM).

Pango lineage	Median p-value	Identified as a VOC / VOI / VUM	Notes (dates given as DD-MM-YYYY)	References
AY.43	7.02E-26		subvariant of Delta (de-escalated VOC as per the ECDC)	https://www.ecdc.europa.eu/en/covid-19/variants-concern
BA.2	4.94E-55		de-escalated VOC as per the ECDC	https://www.ecdc.europa.eu/en/covid-19/variants-concern
BA.2.10.1	0.005404522			
BF.7	2.41E-12		enhanced neutralization resistance respective of parental BA.4/5 subvariant	Qu. P et al., 2023
BF.7.14	6.84E-31			
BN.1.2	1.97E-15			
BN.1.3	2.69E-16			
BQ.1	5.46E-14	VOI (20-10-2022)	VOI as per ECDC (20-10-2022); enhanced neutralization resistance respective of parental BA.5 subvariant	Qu. P et al., 2023; https://www.ecdc.europa.eu/en/publications-data/spread-sars-cov-2-omicron-variant-sub-lineage-bq1-eueea
BQ.1.1	3.25E-25		resistant to all clinical mAbs / enhanced neutralization resistance respective of parental BA.5 subvariant	Arora P., et al. 2022; Qu P., et al., 2023
BR.2.1	6.67E-159			
CH.1.1	0.026651959	VUM (08-02-2023)	WHO variant under monitoring since (08-02-2023)	https://www.who.int/activities/tracking-SARS-CoV-2-variants
CH.1.1.1	0.000159952			
DY.2	5.38E-08			
EG.1	2.85E-08			
EG.2	0.00019372			
EL.1	0.012517337			
FE.1	8.68E-07	VUM	VUM as per ECDC	https://www.ecdc.europa.eu/en/covid-19/variants-concern
FE.1.2	4.23E-25			
FL.2	1.08E-22			
FR.1	0.020160177			
Unassigned	0.029477699			
XBB	1.42E-58	VUM (12-10-2022)	XBB and its sublineages have been identified as variants under monitoring as per the WHO (12-10-2022)	https://www.who.int/activities/tracking-SARS-CoV-2-variants
XBB.1	1.60E-05			
XBB.1.15	0.002277383	VOI (11-01-2023)	WHO current variant of interest (as of 11-01-2023)	https://www.who.int/activities/tracking-SARS-CoV-2-variants

XBB.1.16	5.97E-10	VOI (17-04-2023)	WHO current variant of interest (as of 17-04-2023)	https://www.who.int/activities/tracking-SARS-CoV-2-variants
XBB.1.16.1	0.006501881			
XBB.1.18.1	2.35E-22			
XBB.1.5	4.52E-22			
XBB.1.5.12	2.84E-05			
XBB.1.5.25	3.96E-41			
XBB.1.5.33	5.67E-05			
XBB.1.9.1	3.02E-05	VUM (30-03-2023)	WHO variant under monitoring since (30-03-2023)	https://www.who.int/activities/tracking-SARS-CoV-2-variants
XBB.1.9.2	2.18E-07	VUM (26-04-2023)	WHO variant under monitoring since (26-04-2023)	https://www.who.int/activities/tracking-SARS-CoV-2-variants
XBB.2	8.09E-58			
XBB.2.3.2	6.99E-10			
XBB.2.6	1.24E-115			
XBF	2.68E-08		increased antibody neutralization	Ackerman A., et al. 2023
