# Supplementary material for: In silico genomic surveillance by CoVerage predicts and characterizes SARS-CoV-2 variants of interest
Source: Nat Commun. 2025 Jul 8;16:6281. doi: 10.1038/s41467-025-60231-4 (PMC12238648; doi:10.1038/s41467-025-60231-4)
Supplement: Supplementary file 2 — Reporting Summary [file 41467_2025_60231_MOESM2_ESM.pdf]

nature portfolio

Corresponding author(s): Alice C. McHardy

Last updated by author(s): 2025/05/05

Reporting Summary

Nature Portfolio wishes to improve the reproducibility of the work that we publish. This form provides structure for consistency and transparency in reporting. For further information on Nature Portfolio policies, see our Editorial Policies and the Editorial Policy Checklist.

Statistics

For all statistical analyses, confirm that the following items are present in the figure legend, table legend, main text, or Methods section.

n/a	Confirmed	
☐	☒	The exact sample size (*n*) for each experimental group/condition, given as a discrete number and unit of measurement
☒	☐	A statement on whether measurements were taken from distinct samples or whether the same sample was measured repeatedly
☐	☒	The statistical test(s) used AND whether they are one- or two-sided<br>*Only common tests should be described solely by name; describe more complex techniques in the Methods section.*
☐	☒	A description of all covariates tested
☐	☒	A description of any assumptions or corrections, such as tests of normality and adjustment for multiple comparisons
☐	☒	A full description of the statistical parameters including central tendency (e.g. means) or other basic estimates (e.g. regression coefficient) AND variation (e.g. standard deviation) or associated estimates of uncertainty (e.g. confidence intervals)
☐	☒	For null hypothesis testing, the test statistic (e.g. *F*, *t*, *r*) with confidence intervals, effect sizes, degrees of freedom and *P* value noted<br>*Give P values as exact values whenever suitable.*
☒	☐	For Bayesian analysis, information on the choice of priors and Markov chain Monte Carlo settings
☒	☐	For hierarchical and complex designs, identification of the appropriate level for tests and full reporting of outcomes
☒	☐	Estimates of effect sizes (e.g. Cohen's *d*, Pearson's *r*), indicating how they were calculated

Our web collection on statistics for biologists contains articles on many of the points above.

Software and code

Policy information about availability of computer code

Data collection	Data was obtained from the public repository GISAID (http://gisaid.org), and the accession IDs used are available on the following GitHub repositories:<br>https://github.com/hzi-bifo/corona_lineage_dynamics/tree/main/data<br>https://github.com/hzi-bifo/Corona_Variant_Scoring/tree/main/data
Data analysis	All code used in this study has been archived on Zenodo and is publicly available:<br>Lineage Dynamics: https://doi.org/10.5281/zenodo.15311135 (GitHub:https://github.com/hzi-bifo/corona_lineage_dynamics)<br>Protein Dynamics: https://doi.org/10.5281/zenodo.15311152 (GitHub: https://github.com/hzi-bifo/corona_protein_dynamics)<br>Variant Scoring: https://doi.org/10.5281/zenodo.15322216 (GitHub: https://github.com/hzi-bifo/Corona_Variant_Scoring)

For manuscripts utilizing custom algorithms or software that are central to the research but not yet described in published literature, software must be made available to editors and reviewers. We strongly encourage code deposition in a community repository (e.g. GitHub). See the Nature Portfolio guidelines for submitting code & software for further information.

Data

Policy information about availability of data

All manuscripts must include a data availability statement. This statement should provide the following information, where applicable:

- Accession codes, unique identifiers, or web links for publicly available datasets
- A description of any restrictions on data availability
- For clinical datasets or third party data, please ensure that the statement adheres to our policy

The results generated in this study for the lineage dynamics and antigenic scoring analyses have been deposited in the Zenodo database under accession code 10171227 (https://zenodo.org/records/10171227). The GISAID genomic data and metadata are available under restricted access as per the GISAID User Agreement prohibiting the distribution of the accessed data, access can be obtained by creating a GISAID account (https://gisaid.org/) and agreeing to the GISAID Database Access Agreement. More information can be found on their FAQ page (https://gisaid.org/help/faq/). The raw genomic data are protected and are not available due to data privacy laws. The processed genomic data and metadata are available on Zenodo at the link provided above. The processed data used for figure generation in this study are provided in the Supplementary Information/Source Data file. The genomic data accession IDs from GISAID used in this study are available in the GitHub databases:
https://github.com/hzi-bifo/corona_lineage_dynamics/tree/main/data
https://github.com/hzi-bifo/Corona_Variant_Scoring/tree/main/data

Research involving human participants, their data, or biological material

Policy information about studies with human participants or human data. See also policy information about sex, gender (identity/presentation), and sexual orientation and race, ethnicity and racism.

Reporting on sex and gender	N/a
Reporting on race, ethnicity, or other socially relevant groupings	N/a
Population characteristics	N/a
Recruitment	Participants were not recruited for this study.
Ethics oversight	N/a

Note that full information on the approval of the study protocol must also be provided in the manuscript.

Field-specific reporting

Please select the one below that is the best fit for your research. If you are not sure, read the appropriate sections before making your selection.

☐ Life sciences ☐ Behavioural & social sciences ☒ Ecological, evolutionary & environmental sciences

For a reference copy of the document with all sections, see nature.com/documents/nr-reporting-summary-flat.pdf

Ecological, evolutionary & environmental sciences study design

All studies must disclose on these points even when the disclosure is negative.

Study description	We used both genomic sequences and metadata as provided by GISAID at different time points to predict potential Variants of Concern for the SARS-CoV-2 virus using novel in-silico methods.
Research sample	The GISAID metadata used, as provided by https://gisaid.org, included isolate accession IDs, collection date, submission date, submitting lab, virus name, Pango lineage, host, and mutation information. The genomic sequences also provided by GISAID contained the genomic sequences of the virus as well as ID and collection date.
Sampling strategy	N/a
Data collection	Data was downloaded from the public repository, GISAID (https://gisaid.org).
Timing and spatial scale	N/a
Data exclusions	Isolates that were marked as "under review" by GISAID were not included in this study as these sequences were either representative of SARS-CoV-2 lineages that arose too early (compared to their WHO first sequenced date).
Reproducibility	Accession IDs for each of the experiments were provided for reproducibility and are available at the following:<br>https://github.com/hzi-bifo/corona_lineage_dynamics/tree/main/data<br>https://github.com/hzi-bifo/Corona_Variant_Scoring/tree/main/data

Randomization	N/a
Blinding	Blinding wasn't relevant to this study.

Did the study involve field work? ☐ Yes ☒ No

Reporting for specific materials, systems and methods

We require information from authors about some types of materials, experimental systems and methods used in many studies. Here, indicate whether each material, system or method listed is relevant to your study. If you are not sure if a list item applies to your research, read the appropriate section before selecting a response.

Materials & experimental systems

n/a	Involved in the study	
☒	☐	Antibodies
☒	☐	Eukaryotic cell lines
☒	☐	Palaeontology and archaeology
☒	☐	Animals and other organisms
☒	☐	Clinical data
☒	☐	Dual use research of concern
☒	☐	Plants

Methods

n/a	Involved in the study	
☒	☐	ChIP-seq
☒	☐	Flow cytometry
☒	☐	MRI-based neuroimaging

Plants

Seed stocks	N/a
Novel plant genotypes	N/a
Authentication	N/a
